# Effects of Composite Probiotic Food on Urinary, Serum Biochemical and Oxidative Stress Parameters in a Urolithiatic Rat Model

**DOI:** 10.17113/ftb.62.03.24.8372

**Published:** 2024-09

**Authors:** V. Sreeja, Deepti Suman, Nasim Vahora, Jashbhai Prajapati

**Affiliations:** Dairy Microbiology Department, SMC College of Dairy Science, Kamdhenu University, Anand-388 110, Gujarat, India

**Keywords:** probiotic food, dietary control of urolithiasis, fermented beverage, milk and barley, antiurolithiatic properties

## Abstract

**Research background:**

Prevention, management or cure of diseases through dietary approaches is becoming increasingly important. Research suggests that probiotic, oxalate-degrading *Lactobacillus* species administered *via* a milk and cereal food can prevent kidney stones while also addressing nutritional deficiencies and maintaining essential calcium levels. This study investigates the effect of a composite probiotic milk beverage on urolithiatic rats.

**Experimental approach:**

Probiotic milk-barley beverage (PMBB) was prepared by fermentation of milk enriched with barley (*Hordeum vulgare*) flour using starter culture containing oxalate-degrading probiotic strains (*Lacticaseibacillus rhamnosus* strains MTCC5945 and MTCC25062, *Lactobacillus helveticus* MTCC5463 and *Lactiplantibacillus plantarum* M11). Cumin and common salt were used as flavourings. Unfermented milk-barley base (C) served as control. Wistar rats were divided in four groups (*N*=6). Normal control (NC) group received normal rat diet, and to induce kidney stones, ethylene glycol (0.75 %) and ammonium chloride (1 %) were administered to the disease control (DC) group, PMBB and control (C) groups for 28 days. PMBB and C groups received 1 mL of probiotic milk and barley beverage and unfermented milk-barley base from day 15 to day 28. Indicators of urolithiasis were studied.

**Results and conclusions:**

PMBB significantly (p<0.05) increased urine output, decreased urine oxalate concentrations and increased creatinine, calcium and uric acid concentrations. Serum parameters such as concentrations of calcium, uric acid, urea and creatinine increased significantly (p<0.05) in DC rats, but decreased significantly (p<0.05) in the PMBB group. In addition, serum concentrations of magnesium and osteopontin decreased more significantly in DC rats than in the PMBB ones. The increase in malondialdehyde and decrease in reduced glutathione concentrations observed in the DC group were significantly lower in the PMBB group. The histomorphology of the kidney tissue of DC rats showed calcium oxalate crystal aggregates in the tubules, indicators of renal injury, tubular dilatation, enlarged urinary space and shrunken glomeruli. The PMBB group showed an improvement in the renal histological architecture. Analysis of the caecal digesta of the rats showed a significantly (p<0.05) higher mass fraction of fatty acids (acetic and propionic) in the treatment group. The results show the potential of PMBB in the dietary control of urolithiasis.

**Novelty and scientific contribution:**

This study focuses on the antiurolithiatic prospect of a composite probiotic milk beverage produced with oxalate-degrading culture. It also points to a new functional food form that shows promising health benefits in nutrition of patients with urolithiasis.

## INTRODUCTION

Dietary approaches to prevent, treat or cure diseases are becoming more and more important. Food products with specific health-promoting properties are the need of the hour. To meet this challenge, natural food ingredients with the desired nutritional and functional properties must be used and such ingredients must be combined to develop functional food products and validate the potential health benefits through studies.

Urolithiasis is a urological disorder that affects all age groups, genders and races. Its prevalence is reported to be increasing. About 80 % of people who develop urolithiasis have calcium oxalate stones. Oxalate buildup is an important factor in this disease. In addition to the genetic predisposition of the host, socioeconomic status, environment, lack of physical activity and food choices also contribute to the increase of this disease (*1*, *2*). In addition, studies on gut microbiome suggest that gut microbiota dysbiosis may play a vital role in the increasing prevalence of urolithiasis (*2*, *3*). Pharmaceutical and home treatments for urinary stones cannot completely eliminate the risk of developing new stones. Although the removal of urinary stones has improved significantly thanks to technological breakthroughs, several of these techniques also have drawbacks, such as the high cost of procedures for the average person, stone recurrence and a number of negative side effects (*4*). In addition, dietary restriction approaches are not considered good as they can lead to nutritional deficiencies. Moreover, diet is considered to play a crucial role in the overall medical approach to urolithiasis and it also plays a fundamental role in the composition and functionality of human gut microbiota (*3*). Therefore, dietary measures offer a possible way to treat microbiota-related diseases and kidney stone formation.

In the context of the involvement of a dysbiotic gut microbiota in the progression of urolithiasis, it has been reported that bacteria in the gut that metabolise oxalate minimise free soluble oxalate by degrading it to formic acid and CO_2_, and thus preventing its absorption (*5*-*7*). *Oxalobacter formigenes*, which lives in the human gut, is a known oxalate-degrading bacterium but a poor intestinal coloniser (*8*). Different studies have shown the ability of lactic acid bacteria (LAB), particularly of genus *Lactobacillus*, to degrade intestinal oxalate (*6*, *9*-*13*). Patients with urolithiasis have been reported to have a decrease in oxaluria when treated with an oral mixture of freeze-dried, oxalate-degrading LAB (*14*). Turroni *et al*. (*9*) reported that many probiotic *Lactobacillus* sp. were capable of degrading oxalate to a great extent. Studies in animal models of primary hyperoxaluria have shown that selected *Lactobacillus* sp. were able to reduce urinary oxalate. Although the health benefits of *Lactobacillus* probiotics vary greatly depending on the species, there is evidence for their potential use as probiotics to treat hyperoxaluria (*15*, *16*). A recombinant strain of *Lb. plantarum* was able to degrade up to 90 % oxalate compared to 15 % by a non-modified strain (*17*). As the use of genetically modified organisms is associated with legal hurdles, the search for food-derived LAB that can degrade oxalate still continues. According to Stepanova *et al*. (*16*) the oxalate content in urine and the accumulation of calcium oxalate in kidney tissue decreased when oxalate-utilising bacteria were used with herbal extracts.

Hypercalciuria is considered one of the dangers for kidney stone formation. Therefore, it used to be common practice to ask people with calcium stones to avoid a calcium-rich diet. However, it has now been shown that a lower calcium intake can lead to increased absorption of oxalate in the intestine and thus to an increased risk of kidney stone formation (*1*). In addition, a diet with sufficient calcium and less oxalate is considered the usual therapy for people with mild hyperoxaluria. It has also been suggested that calcium should preferably be obtained from foods such as milk products, since its supplements may slightly increase the risk of stone formation (*12*). Therefore, taking calcium through a milk-based beverage can be a better option. In addition, such foods can compensate for the nutritional deficiencies associated with urolithiasis.

Barley is considered a diuretic among cereals, and the barley water, which is obtained after cooking the grain, is traditionally considered very beneficial for all urinary and kidney disease conditions. An alkaline preparation of barley has been reported to be effective in dissolving kidney stones and improving symptoms (*18*). Furthermore, β-glucans in barley grains have been reported to reduce chronic kidney disease (*19*). Shah *et al*. (*20*) found that the administration of barley extract to calculogenic rats reduced and prevented the growth of stones and that the therapeutic effect was better than the preventive effect. They attributed this effect to the diuretic potential, antioxidant activity, nephroprotective property and the ability of barley extract to reduce the concentration of components responsible for stone formation. Barley has been reported to be a rich source of nutrients and functional ingredients that have antioxidant, antiobesity, antiproliferative, anticancer, antidiabetes and cholesterol-lowering potential (*19*, *21*).

This study is the first of its kind to investigate the effect of a novel composite probiotic milk beverage made with oxalate-degrading LAB strains on kidney stones.

## MATERIALS AND METHODS

### LAB strains

The starter culture consisted of the oxalate-degrading strains *Lacticaseibacillus rhamnosus* strains MTCC 5945 and MTCC 25062, the probiotic *Lactobacillus helveticus* MTCC 5463 and *Lactiplantibacillus plantarum* M11 (Table 1). The strains were previously studied for their ability to degrade oxalate (*22*), their probiotic properties and also for the presence of *oxc* (encoding oxalyl-coenzyme A decarboxylase) by a molecular study (not part of this manuscript). Among the four strains, only MTCC 5463 showed the presence of *oxc*. All the strains were procured from the culture collection of SMC College of Dairy Science, Anand, Gujarat, India. The LAB strains were kept active throughout the study by propagating them in sterile skimmed milk medium (11 % total solids) by incubation ((37±1) °C for 12 h) and stored at (7±1) °C.

**Table 1:** Details of *Lactobacillus* strains used in the study

Strain	Source of isolation	Accession number	Oxalate degradation/%
*Lacticaseibacillus rhamnosus* MTCC 5945	Traditional curd	KJ156963	49.2
*Lacticaseibacillus rhamnosus* MTCC 25062	Traditional curd	KR732326	56.5
*Lactobacillus helveticus* MTCC 5463	Human vagina	GQ253959	68.8
*Lactiplantibacillus plantarum* M11	Traditional curd	SAMN11890673	69.7

### Composite food

The composite food used in this study was a probiotic milk-barley beverage (PMBB). Unfermented milk-barley base served as control (C).

#### Preparation of PMBB and its analysis

PMBB was made from milk (11.5 % total solids) and barley water. For the preparation of barley water, barley (*Hordeum vulgare*) flour (10 g, purchased from the local market, Anand, Gujarat, India) was sifted to remove any foreign matter. It was then dissolved in lukewarm water (45 °C) in the ratio of 10:90. The mixture was then boiled for 10 min with constant stirring and then cooled and stored at (7±1) °C. The barley water thus obtained was added to the milk to achieve 4 g barley in 100 mL mixture. The mixture was heated to 85 °C for 5 min with constant stirring and cooled to 37 °C before the 2 % starter culture (consisting of MTCC 5463, MTCC 5945, MTCC 25062 and M11) was added. Incubation ((37±1) °C) was carried out until the titratable acidity reached 0.65–0.70 % lactic acid. The coagulum was cooled and further stirred to get a homogeneous beverage. Roasted cumin powder at 0.5 % and common salt at 0.4 % were added as flavourings. The beverage was stored in high density polyethylene bottles at (7±1) °C.

The pH, *Lactobacillus* count, overall sensory acceptability (rating on the hedonic scale), total solids, fat and calcium content of the probiotic beverage were analysed using standard methods. To determine the titratable acidity, 10 mL of product sample were titrated with 0.1 M NaOH using 1 % (*m*/*V*) phenolphthalein (Loba Chemicals, Ahmedabad, India) as an indicator (*23*). A pH meter (Oakton pH 700; MSN Enterprise, Haryana, India) was used to measure the pH at 25 °C.

The lactic acid bacteria were enumerated according to the method of Chaudhary and Mudgal (*24*). Briefly, 1 mL of the beverage was aseptically added to 9 mL of sterile phosphate buffer (HiMedia, Mumbai, India) to obtain the first dilution. The further dilutions of 8 and 9 times were prepared in phosphate buffer and a volume of 1 mL was pour plated in duplicate Petri plates containing MRS agar (HiMedia). The double layering method was used. The samples were incubated at (37±1) °C for 72 h. Typical colonies of lactic acid bacteria on the plates were counted and the number was expressed as log CFU/mL (*24*).

The sensory acceptability (flavour, body and texture, colour and appearance, and overall acceptability criteria) of the probiotic beverages was evaluated by a panel of judges (*N*=10) aged 25 to 50, consisting of equal number of men and women with experience in sensory evaluation using a 9-point hedonic scale (*25*).

Fat and total solids in PMBB were determined using Mojonnier and gravimetric methods respectively (*26*). For this purpose, 1 g of PMBB was mixed with 10 mL of concentrated hydrochloric acid (Loba Chemicals) and the mixture was heated in a water bath until the proteins had dissolved. The content was cooled and transferred to a Mojonnier flask with 10 mL of alcohol. A volume of 25 mL each of diethyl ether (Merck, Bangalore, India) and petroleum ether (Merck) was added. The contents were mixed and allowed to stand until the two layers separated completely. The upper layer was transferred to a weighed beaker. The extraction was repeated twice with 15 mL of the solvent each time. The solvent was completely evaporated in a water bath. The beaker was heated in an oven at (100±1) °C for 1 h, cooled in a desiccator and weighed. The mass fraction of fat was calculated as follows:


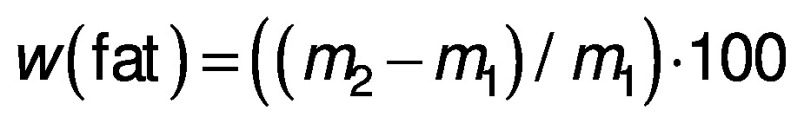
 /1/

where *m*_1_ is the mass of beverage and *m*_2_ is the mass of conical flask and residue after drying.

To determine the total solids, 5 g of the beverage was dried in a boiling water bath and then in a hot air oven ((102±2) °C) to obtain the residue. The total solids content was determined as follows:


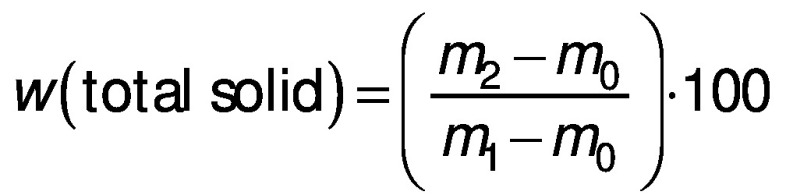
 /2/

where *m*_0_ is the mass (g) of dish and lid, *m*_1_ is the sum of *m*_0_ and *m*_sample_ and *m*_2_ is the sum of *m*_0_ and *m*_dried sample_.

Calcium mass fraction in PMBB was determined according to the Bureau of Indian Standards IS 1479-2 method (*27*). Briefly, 25 mL of ash dissolved in hydrochloric acid were pipetted into a 250-mL beaker and diluted to about 50 mL with distilled water. Methyl red indicator (HiMedia) was added and the pH was adjusted to 5.6 with ammonium hydroxide (SD Fine–Chem Ltd., Mumbai, India). To this, 10 mL of saturated ammonium oxalate (Merck) was added until precipitates formed. The precipitates were dissolved in 25 mL of 1:4 diluted sulfuric acid (Loba Chemicals) and titrated against 0.002 M potassium permanganate (Loba Chemicals) until a permanent pink colour appeared. Calcium was determined in mg/100 g as follows:


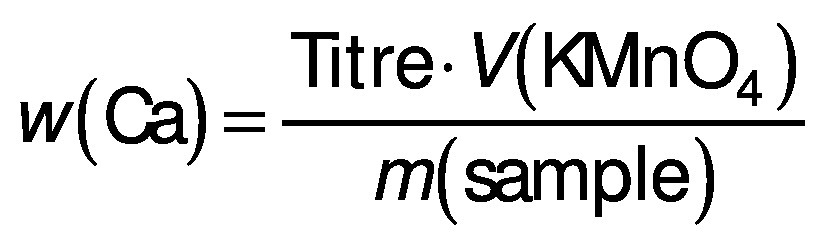
 /3/

where titre=1.002 g/mL.

### Animal model and experimental design

Institutional Animal Ethics Committee (IAEC) approved the experimental design. Wistar rats were handled according to the guidelines of the Committee for the Purpose of Control and Supervision of Experiments on Animals (CPCSEA), Ministry of Social Justice and Empowerment, Government of India (Protocol No: RPCP/IAEC/2018–2019/R37). The experimental rats (Zydus Research Center, Ahmedabad, Gujarat, India) were housed in polypropylene rat cages (three rats per cage) and were acclimatised to standard conditions ((25±2) °C, 12 h light/12 h dark cycle) throughout the study. The rats received pellet diet and water *ad libitum*.

Male Wistar albino rats (200–280 g) were used in four groups of six animals each. Normal control (NC) group received normal rat diet and drinking water *ad libitum*. To induce kidney stone formation, 0.75 % ethylene glycol and 1 % ammonium chloride (Sigma-Aldrich, Merck, Bangalore, India) were administered in the drinking water to the disease control (DC) group and the treatment groups PMBB and C. DC group received ethylene glycol and ammonium chloride for 28 days. PMBB and C groups received fermented probiotic beverage and unfermented milk and barley base respectively, from day 15 to day 28. The products were administered orally to the rats (1 mL/day).

### Assessment of antiurolithiatic activity

#### Urine analysis

The experimental rats were kept individually in metabolic cages in which urine was collected for 24 h. The urine samples were analysed for calcium, creatinine, uric acid, oxalate and citrate content. Urinary calcium, uric acid and creatinine concentrations were determined using the autoanalyzer (Chemray, Rayto Life and Analytical Sciences Co., Ltd, Shenzhen, PR China). Urinary oxalate was measured according to Hodgkinson (*28*). Urine pH (2 mL) was adjusted to 5.0–5.2 by adding either ammonia or acetic acid, followed by the addition of 0.2 mL of calcium chloride (HiMedia) to each sample and the mixture was kept at room temperature for 16 h. The calcium oxalate precipitates were separated by centrifugation, cleaned and dissolved in 0.5 M sulfuric acid and titrated against 0.002 M potassium permanganate (HiMedia). Briefly, for the estimation of urinary citrate, 0.2 mL of sodium potassium tartrate (HiMedia) solution was added to an aqueous 0.8 mL solution of cupric chloride (Merck), which was then mixed with 0.2 mL of urine sample. The formation of a blue coloured complex within 10 min was measured at 760 nm (BL 222 double beam bio spectrophotometer; Elico, Hyderabad, India) The citrate content was expressed in mg per dL of urine collected for 24 h.

#### Serum analysis

Blood was collected at the end of the 28-day study by retro-orbital sampling under anaesthetic conditions and the rats were sacrificed. Serum was collected by centrifugation (R-8C BL bench top centrifuge; Remi Elektrotechnik Limited, Mumbai, India) at 2500×*g* for 10 min. Calcium, magnesium, phosphorus, uric acid, urea and creatinine concentrations of the serum were determined using autoanalyzer (Chemray, Rayto Life and Analytical Sciences Co., Ltd). Serum osteopontin and uromodulin were analysed using ELISA kit (ELK, Wuhan Biotech Ltd., PR China) according to the manufacturer’s instructions.

#### Analysis of kidney tissue homogenate

A mass of 1 g of kidney tissue homogenate obtained by homogenization (Remi digital high speed homogenizer (RQT 127 A/D); REMI Sales & Engineering Ltd., Ahmedabad, India) in phosphate-buffered saline (pH=7.4) was centrifuged (R-8C BL Bench top centrifuge; Remi Elektrotechnik Ltd, Mumbai, India) at 3920×*g* for 20 min and the obtained supernatant was used for the determination of reduced glutathione (GSH), malondialdehyde (MDA) and superoxide dismutase (SOD). For the determination of malondialdehyde, 0.5 mL of the sample and 0.5 mL of Tris HCL were incubated at 37 °C for 2 h and then 1 mL of 10 % trichloroacetic acid (HiMedia) was added and centrifuged (R-8C BL bench top centrifuge; Remi Elektrotechnik Ltd) at 157×*g* for 10 min to obtain the supernatant. A volume of 1 mL of 0.67 % thiobarbituric acid (Merck) was added to 1 mL of the supernatant and kept in boiling water for 10 min. Distilled water (1 mL) was then added and the absorbance was measured at 532 nm (PC-based double beam UV-Vis spectrophotometer 2206; Systronics, Ahmedabad, India). The results were expressed in nmol of malondialdehyde per g of tissue.

Mass fraction of reduced GSH in the kidney tissue homogenate (1 g) was measured as follows. In brief, 1 mL of supernatant was obtained with 1 mL of 4 % sulfosalicylic acid (Merck) by incubation at 4 °C for 1 h and refrigerated centrifugation (R-8C BL bench top centrifuge; Remi Elektrotechnik Ltd) at 226×*g* for 15 min. The supernatant (1 mL) was mixed with 0.1 M phosphate buffer (2.7 mL, pH=7.4) and 0.2 mL 5,5-dithiobis-2-nitrobenzoic acid (HiMedia). The developed yellow colour was measured using the spectrophotometer (PC-based double beam UV-Vis spectrophotometer 2206; Systronics) at 412 nm. The results were expressed as μmol GSH per g of tissue.

The activity of SOD in the kidney homogenate was measured as follows. Briefly, the reaction medium consisted of 50 mM Tris-Cl buffer (HiMedia), pH=8.2, and 1 mM EDTA (Merck). Tissue homogenate (2 mL) was mixed with 0.2 mM pyrogallol (HiMedia) to start the reaction and the absorbance was measured at 420 nm (PC-based double beam UV-Vis spectrophotometer 2206; Systronics). The percentage of inhibition of pyrogallol autoxidation was calculated:


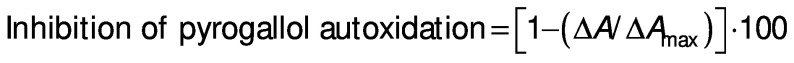
 /4/

where Δ*A* is the absorbance as a result of pyrogallol autoxidation in the sample and Δ*A*_max_ is the absorbance as a result of pyrogallol autoxidation in the control (without cell lysate).

### Enumeration of the faecal Lactobacillus count

The procedure of Chaudhary and Mudgal (*24*) was followed. Briefly, faecal samples were diluted 7, 8 and 9 times and pour plated on sterile, *Lactobacillus* MRS agar. Typical colonies of lactobacilli were counted after incubation (37 °C for 72 h) and the result was expressed in log CFU/g.

### Estimation of short-chain fatty acid content in the caecal matter

The short-chain fatty acids (SCFAs) in the caecal matter were estimated according to Asano *et al*. (*29*). Briefly, 10 mL of 0.05 M sulfuric acid (HiMedia) was added to 1 g of cecal content and the mixture was homogenised and centrifuged (R-8C BL bench top centrifuge; Remi Elektrotechnik Ltd) at 4240×*g* for 10 min. The supernatant was filtered (0.45 µm membrane filter) and injected into the HPLC (LC-20; Shimadzu, Tokyo, Japan) using a microinjector (HAMILTON Bonaduz AG, Bonaduz, Switzerland) with a 20 µL loop. Discovery® BIO Wide Pore C18 analytical column (SeQuant® ZIC®-cHILIC, 3 µ, 250 mm×4.6 mm; MZ-Analysentechnik GmbH, Mainz, Germany) was used. The samples were eluted with an isocratic mixture of 0.1 % (*V*/*V*) phosphoric acid at 30 °C and a flow rate of 0.7 mL/min. A UV detector (SPD-20A UV/Vis detector; Shimadzu) at 210 nm was used. The SCFA (butyrate, acetate and propionate) in the caecal samples were determined for all groups.

### Histopathological analysis

Samples of kidney tissue, fixed in 10 % buffered formalin (Merck) and embedded in paraffin, were sectioned with a microtome (5 μm thickness) and fixed on slides. Slides stained with haematoxylin and eosin (Merck) were observed using a trinocular microscope (Olympus Corporation, Tokyo, Japan).

### Statistical analysis

The data were expressed as mean value±standard deviation and statistically analysed by one-way ANOVA followed by Tukey's multiple range test using GraphPad Prism v. 8.00. (*30*). Values at p<0.05 were considered significantly different.

## RESULTS AND DISCUSSION

In addition to genetic predisposition and lifestyle changes, dietary factors and the lack of oxalate utilising microbes in the gut are also cited as factors that cause kidney stones. It would therefore be interesting to know what effect a probiotic milk and barley beverage prepared with oxalate-degrading strains has on urolithiatic changes. Researchers have reported on the potential of probiotic *Lactobacillus* strains to degrade oxalate in the prevention of kidney stones (*6*, *9*, *14*, *31*). Most of these studies have used freeze-dried preparations of probiotic strains. We could not find any previous studies that investigated the ameliorating effect of a composite probiotic beverage against kidney stones. As far as we know, this study is the first to examine this topic.

In this study we investigated the effect of oral administration of PMBB on urinary, serum biochemical and oxidative stress parameters in ethylene glycol- and ammonium chloride-induced kidney stones in a male Wistar rat model. The oxalate-degrading potential of the strains we used in this study ranged from 49.2 to 69.7 %, and only MTCC 5463 had the *oxc* gene. The antioxidant activity of PMBB was (86.13±0.06) %, measured as ABTS radical scavenging activity (results not part of this manuscript). Since PMBB is a probiotic food, its sensory acceptability and probiotic count are crucial. During the experiment, the probiotic count in PMBB was (9.4±0.2) CFU/mL and the pH was 4.72±0.08 (data not shown). The overall sensory acceptability score of PMBB on the hedonic scale (out of 9) was 8.05±0.07 (like very much), as determined by the sensory panel experts (data not shown). Total solids, fat and calcium mass fractions of the product were (11.19±0.09), (1.72±0.03) and (72.3±1.5) mg/100 g, respectively (data not shown).

The urine of most people is reported to be supersaturated, which favours the crystallisation of calcium oxalate (CaC_2_O_4_) and leads to 70 % or more kidney stones made of CaC_2_O_4_ (*12*). We used male Wistar rats as experimental models because their urinary system is similar to that of humans (*32*). Urolithiasis was induced in rats receiving ethylene glycol and ammonium chloride. Ethylene glycol is reported to be an oxalate precursor and ammonium chloride accelerates lithiasis. This urolithiatic model is commonly used to mimic the development of kidney stones in humans (*32*, *33*).

### Effect of PMBB on urinary parameters

**Fig. 1:**
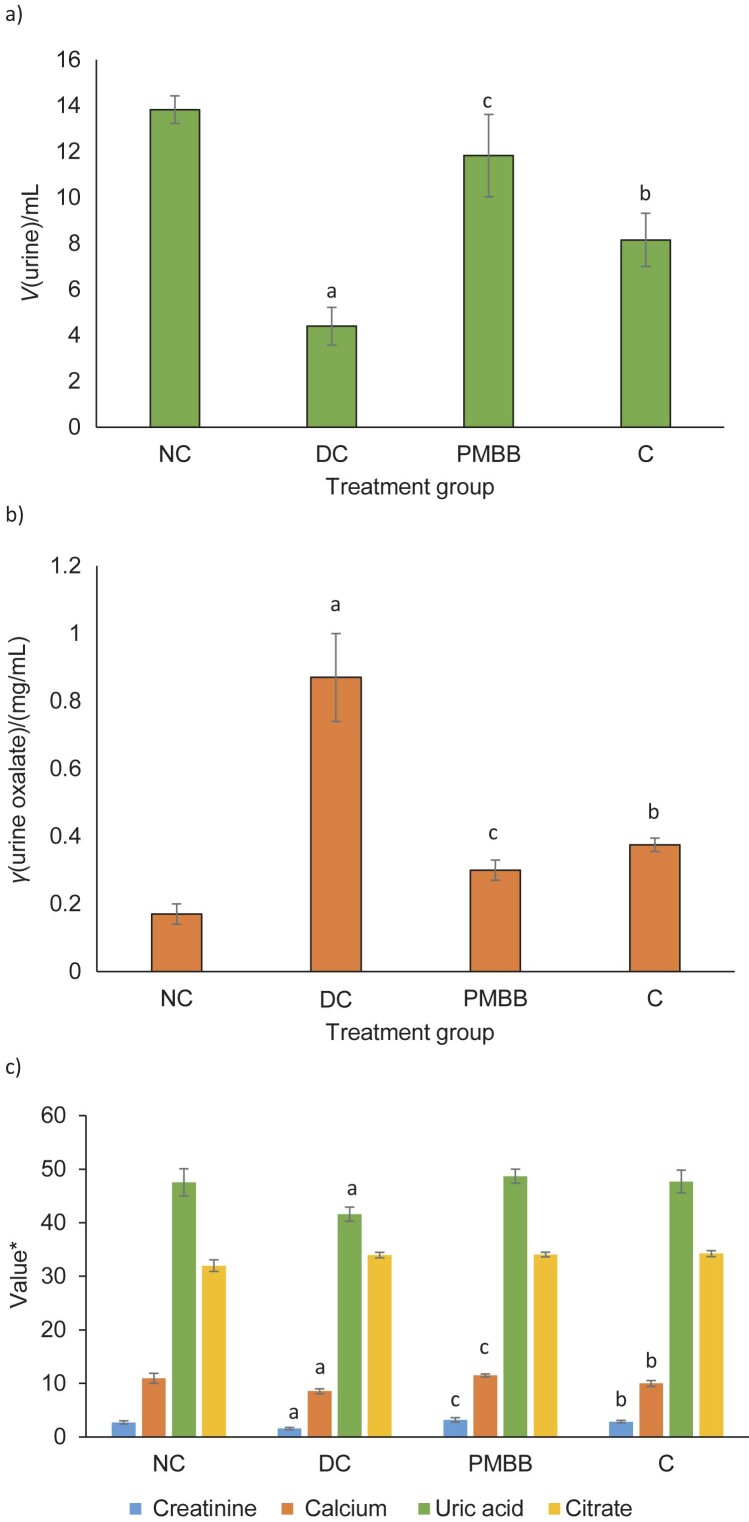
The effect of PMBB on: a) urine output, b) urine oxalate and c) other urinary parameters in the urine of experimental rats collected for 24 h. The results are expressed as mean value±standard deviation (*N*=6). a=values are significant (p<0.05) compared to NC, b=significant (p<0.05) compared to DC, c=significant (p<0.05) compared to C. NC=normal control, DC=disease control, PMBB=rats fed with fermented probiotic milk and barley beverage, C=rats fed with unfermented milk and barley control. *Creatinine, calcium and uric acid concentrations are expressed in mg/dL and citrate is expressed in mg/mL

The effects of the administration of PMBB on urinary parameters are shown in Fig. 1. The administration of ethylene glycol and ammonium chloride for 28 days significantly reduced the urine output, as well as creatinine, uric acid and calcium concentrations in the urine of the DC group compared to the NC group. In addition, a significant (p<0.05) increase in the urine oxalate concentration was observed in the DC rats compared to the NC group. The PMBB and C groups showed a significant (p<0.05) improvement in urinary output compared to the DC group and the effect was highly significant for the PMBB group (Fig. 1a). A significant (p<0.05) decrease in urine oxalate (Fig. 1b) and an increase in creatinine, calcium and uric acid concentrations (Fig. 1c) were also observed in the PMBB and C groups. However, this increase was not significant compared to NC group, suggesting that the change may not be an adverse one. The treatment with fermented probiotic beverage showed a significantly (p<0.05) better effect than the treatment with unfermented milk and barley base. The difference in urinary citrate concentrations among the four groups was not significant.

In this study, the administration of ethylene glycol and ammonium chloride resulted in a significant (p<0.05) decrease in the urine volume and an increase in the urine oxalate concentration (Fig. 1). Our results are consistent with those of previous experiments showing that the administration of ethylene glycol with or without ammonium chloride in drinking water consistently led to the induction of mainly calcium oxalate urolithiasis (*34*-*36*). Treatment with PMBB significantly increased the urine output (Fig. 1a) and decreased the urine oxalate concentration compared to the DC group (Fig. 1b), suggesting that PMBB has a diuretic effect. This increased urine volume may inhibit crystal formation and facilitate the excretion of crystals. It may also dilute the concentration of electrolytes in the urine and reduce the chances of stone formation. Experiments on both animals and humans have shown the effect of administering probiotics on oxalate concentrations in urine. The study by Murphy *et al*. (*31*) on the oxalate-degrading ability of probiotics *in vivo* in a rat model reported that rats given the probiotic strains *Lactobacillus animalis* 223C and *L. animalis* 5323 had lower urinary oxalate excretion. Campieri *et al*. (*14*), in their study on the effect of oral administration of LAB in patients suffering from calcium oxalate urolithiasis, reported a significant decrease in 24-hour excretion of oxalate in patients who received a freeze-dried mixture of lactic acid bacteria (consisting of *L. acidophilus, L. plantarum, L. brevis* and *S. thermophilus)* daily for four weeks. Lieske (*12*) reported on the effect of the probiotic Oxadrop® granulate containing *L. acidophilus*, *L. brevis*, *S. thermophilus* and *B. infantis* against hyperoxaluria. The study by Murru *et al*. (*13*) showed that the administration of Oxadrop® to kidney stone patients led to a reduction in urinary oxalate excretion of almost 18 % after 30 days. However, the mechanism of LAB in reducing urinary oxalate excretion is still unclear. In our study, a 65 % decrease in the urinary oxalate concentration was observed in the PMBB group, while the control group showed 58 % decrease, suggesting that the milk and barley base also plays a crucial role in oxalate excretion. Furthermore, we observed a decrease in calcium, creatinine and uric acid concentrations in the urine of the DC group compared to the NC group. These concentrations were changed after the treatment with PMBB to a similar concentration as in the NC group (Fig. 1c). Our observation was consistent with an experiment by Fan *et al*. (*37*) in which they investigated the effect of administering different amounts of ethylene glycol and ammonium chloride to rats. They reported a decreased calcium excretion and no change in uric acid excretion after administration of 0.75 % ethylene glycol and 1 % ammonium chloride. They also showed that the increased acidification of urine due to the use of ammonium chloride decreased citrate excretion, which may have led to increased deposition of CaC_2_O_4_ crystals in the kidneys. Citrate has been reported to inhibit nucleation of calcium oxalate (*38*). Although not significant, we observed increased citrate concentrations in the PMBB and C groups, indicating the antiurolithiatic activity of the products. Shah *et al*. (*20*) in their study on the curative and preventive effect of *Hordeum vulgare* extract against urolithiasis reported increased urine output, significantly decreased excretion of oxalate, calcium, magnesium, phosphate, urea and uric acid and increased excretion of citrate compared to control rats.

### Effect of PMBB on serum parameters

The DC group showed a significant (p<0.05) increase in serum calcium, creatinine, urea and uric acid concentrations, while serum magnesium, osteopontin and uromodulin concentrations decreased significantly (Table 2). These increased concentrations of calcium, creatinine, urea and uric acid decreased significantly (p<0.05) by treatment with fermented probiotic beverage and unfermented milk and barley base. In addition, the treatment with those two types of drinks led to a significant increase in serum magnesium, osteopontin and uromodulin. Moreover, the PMBB group showed a significantly higher serum magnesium concentration compared to group C (Table 2). The difference in serum phosphate and albumin among the groups was not significant.

**Table 2:** Effect of PMBB on serum biochemical parameters

	Experimental group			
Parameter	NC	DC	PMBB	C
*γ*(Ca)/(mg/dL)	(3.9±0.5)^b^	(5.6±0.4)^a^	(4.3±0.4)^b^	(4.2±0.2)^b^
*γ*(Mg)/(mg/dL)	(1.6±0.1)^c^	(0.7±0.2)^a^	(1.6±0.1)^c^	(1.48±0.2)^b^
*γ*(uric acid)/(mg/dL)	(2.0±0.2)^c^	(3.1±0.1)^a^	(2.46±0.06)^b^	(2.51±0.04)^b^
*γ*(creatinine)/(mg/dL)	(0.95±0.02)^b^	(1.16±0.04)^a^	(1.04±0.02)^b^	(0.98±0.01)^b^
*γ*(urea)/(mg/dL)	(34.0±2.4)^b^	(45.4±4.7)^a^	(36.5±0.9)^b^	(35.0±1.3)^b^
*γ*(osteopontin)/(ng/mL)	(2.13±0.07)^b^	(1.0±0.4)^a^	(2.4±0.3)^b^	(2.65±0.1)^b^
*γ*(uromodulin)/(ng/mL)	(2.9±0.4)^a^	(3.4±0.6)^ab^	(4.4±0.4)^b^	(4.7±0.4)^b^
*γ*(phosphate)/(mg/dL)	(2.4±0.3)^a^	(2.5±0.2)^a^	(1.8±0.2)^a^	(2.0±0.1)^a^
*γ*(albumin)/(mg/dL)	(4.4±0.1)^a^	(4.06±0.07)^a^	(4.0±0.2)^a^	(4.4±0.1)^a^

The results are expressed as mean value±standard deviation (*N*=6). Different letters in superscript in the same row indicate statistically significant (p<0.05) differences. NC=normal control, DC=disease control, PMBB=rats fed with fermented probiotic milk and barley beverage, C=rats fed with unfermented milk and barley control

Concentrations of calcium, urea, uric acid, and creatinine in serum increased, while magnesium and osteopontin decreased with the administration of ethylene glycol and ammonium chloride (Table 2). Serum urea, uric acid and creatinine are considered markers of glomerulus and tubule damage and their increased concentrations in serum indicate impaired renal function. These changes were further confirmed by the signs of kidney damage such as shrunken glomeruli, tubular dilatation and enlarged urinary space observed on the histological slides of the DC group. In urolithiatic rats, the glomerular filtration rate was found to decrease due to obstruction of the urine flow by stones. This impairs the ability of the kidneys to remove waste products, particularly nitrogenous ones such as urea, creatinine and uric acid, causing these waste products to accumulate in the blood (*39*, *40*). The reduced concentrations of magnesium and osteopontin may have further favoured the formation of kidney stones, as these substances are thought to prevent the formation of CaC_2_O_4_ crystals and their retention in the kidney. Magnesium prevents the crystallisation of calcium oxalate by binding to it and forming a soluble complex, resulting in a decrease of the concentration that can cause the precipitation of calcium oxalate (*33*, *41*). The PMBB and C groups showed a significant (p<0.05) reduction in the concentrations of creatinine, uric acid, urea and an increase in the concentrations of magnesium and osteopontin compared to the DC group. Compared to the control, PMBB showed a significantly increased serum magnesium concentration, which could be due to the effect of fermentation to improve the bioavailability of minerals (*42*). In their study on rats, Taheri *et al*. (*43*) found that serum creatinine, calcium and hyperoxaluria decreased significantly when *Lactobacillus* and *Bifidobacterium* strains were administered. They also reported improved histopathological features of the renal tissue, which were very similar to those of the positive control group.

### Effects of PMBB on oxidative stress parameters in kidney homogenate

Administration of ethylene glycol and ammonium chloride caused a significant (p<0.05) increase in lipid peroxidation in kidney homogenate, as indicated by the increase in MDA and decrease in GSH in DC rats compared to NC rats. The rats in the PMBB group showed a significant (p<0.05) decrease in MDA and increase in GSH compared to group C. The amounts of superoxide dismutase were not significantly changed in DC or treatment groups.

**Fig. 2:**
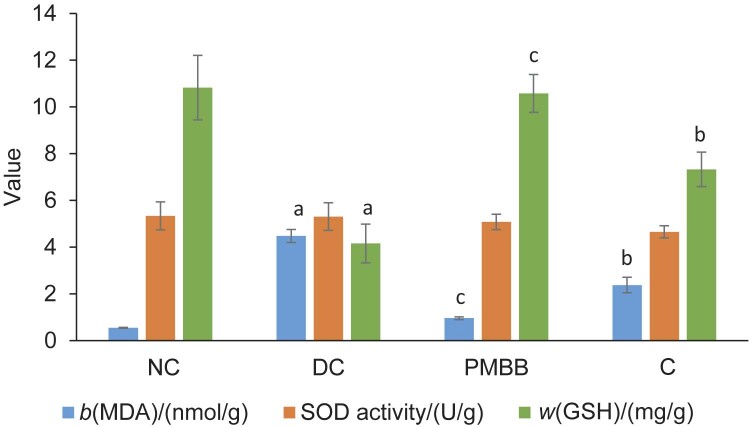
The effect of PMBB on oxidative stress parameters in rats. The results are expressed as mean value±standard deviation (*N*=6). a=significant (p<0.05) compared to NC, b=significant (p<0.05) compared to DC, c=significant (p<0.05) compared to C. NC=normal control, DC=disease control, PMBB=rats fed with fermented probiotic milk and barley beverage, C=rats fed with unfermented probiotic milk and barley control, MDA=malondialdehyde, SOD=superoxide dismutase, GSH=reduced glutathione

The changes in MDA and GSH observed in the DC group (Fig. 2), reflecting the oxidative damage, have been reported to be associated with proximal tubular cell injury. Hirose *et al*. (*44*) reported oxidative stress and renal proximal tubular cell injury in the early phase of kidney crystal formation. An increase in reactive oxygen species (ROS) leads to an increased production of MDA (an oxidative stress biomarker). On the contrary, GSH has a protective role against ROS damage (*45*). Treatment with PMBB caused a significant (p<0.05) decrease in MDA and an increase in GSH activity (Fig. 2). Although not directly related to kidney stones, many studies on other diseases have reported a reduction in oxidative stress markers after supplementation with probiotics. Shah *et al*. (*20*) reported the antioxidant effect of an ethanolic barley extract in reducing the lipid peroxidation in calculogenic rats.

### Effects of PMBB on the SCFA concentration in caecal digesta and the Lactobacillus count in faeces

**Fig. 3:**
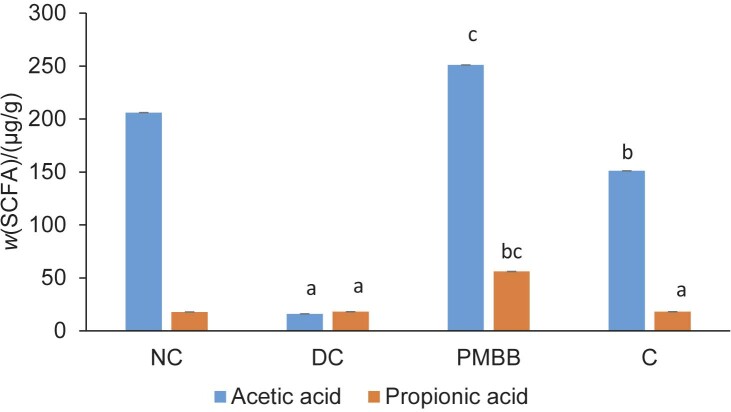
The mass fraction of short-chain fatty acids (SCFA) in the caecal matter of experimental groups. The results are expressed as mean value±standard deviation (*N=*6) a=significant (p<0.05) compared to NC, b=significant (p<0.05) compared to DC, c=significant (p<0.05) compared to C. NC=normal control, DC=disease control, PMBB=rats fed with fermented probiotic milk and barley beverage, C=rats fed with unfermented milk and barley control

The experimental groups differed significantly in their SCFA mass fraction, namely acetic and propionic acids (Fig. 3). We could not detect butyric acid in any of the caecal samples. The PMBB group had significantly (p<0.05) higher mass fractions of acetic and propionic acids than other groups.

*Lactobacillus* count in the faeces of the PMBB group ((9.40±0.03) log CFU/g) was significantly higher, followed by the NC ((8.92±0.05) log CFU/g) and C group ((8.7±0.1) log CFU/g). The lowest count ((7.8±0.2) log CFU/g) was observed in the DC group (data not shown).

Recent studies have shown that short-chain fatty acids (SCFAs) play a potential role in kidney stones. Liu *et al*. (*46*) reported about the relationship between the gut microbiota and SCFAs in renal calcium oxalate stone disease. In model rats, SCFAs (acetate, propionate and butyrate) were shown to attenuate renal calcium oxalate stone formation. Jin *et al*. (*47*) demonstrated the role of immune cells, kidneys and SCFAs in CaC_2_O_4_ formation. In our study, analysis of caecal SCFAs (Fig. 3) showed a higher SCFA mass fraction in the PMBB and C groups. The β-glucans in barley are reported to have prebiotic properties, which may have contributed to this increase. In addition, the higher SCFA in PMBB compared to group C could be due to improved prebiotic utilisation due to fermentation (*48*). Lambo *et al*. (*49*) in their study on the effect of *Lactobacillus* strains on β-glucans in barley reported that fermentation decreased the insoluble fibre content while the soluble fibre remained unchanged. Arena *et al*. (*50*) reported an increased growth of probiotic strains *Lactobacillus acidophilus* LA5, *L. plantarum* WCFS1, *L. plantarum* CETC 8328 and *L. fermentum* CECT 8448 *in vitro* due to the addition of β-glucans. SCFAs have also been reported to play a crucial role in mineral absorption by lowering the pH of the intestinal contents (*51*).

### Histopathological analysis

**Fig. 4:**
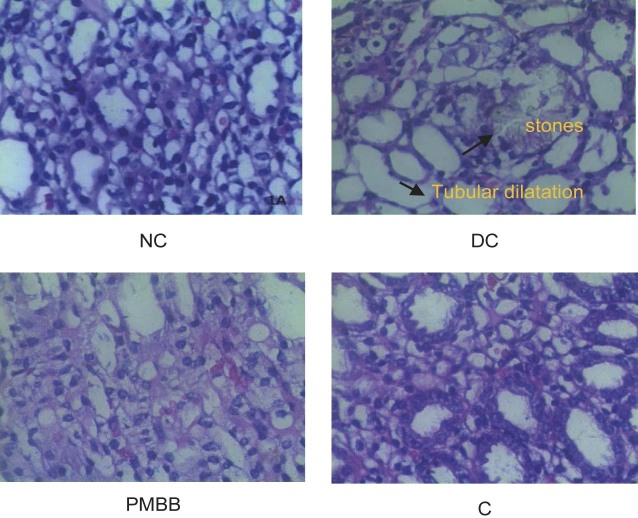
Representative photographs of histopathological analysis of kidney tissue after haematoxylin and eosin staining (60×). NC=normal control, DC=disease control, PMBB=rats fed with fermented probiotic milk and barley beverage, C=rats fed with unfermented milk and barley control

The histomorphological study of kidney tissue of the DC group showed widespread crystal aggregates in the tubules and signs of renal injury such as shrunken glomeruli, dilated tubules and enlarged urinary space (Fig. 4) compared to the NC rats, whose renal histology showed a complete structure of glomeruli, a lobular organisation and a flat epithelial lining of the glomerular capsule. The PMBB and C groups showed an improved renal histological architecture. The renal histology of the rats treated with PMBB was improved much better than that of the C group. The histopathological findings further support the antiurolithiatic activity of PMBB, which recovered compared to the DC group (Fig. 4). This indicates a probable renoprotective effect of the probiotic milk and barley beverage.

## CONCLUSIONS

Overall, the results of our study showed that oral administration of the probiotic dietary preparation improved the changes associated with kidney stone in rats treated with ethylene glycol and ammonium chloride. The composite probiotic milk beverage produced with oxalate-degrading culture showed a better effect on the excretion of creatinine, calcium and oxalate in urine than the unfermented control. It also provided much better protection against oxidative stress, as indicated by its effect on malondialdehyde and reduced glutathione mass fraction compared to the control. The results of this study suggest that the composite probiotic beverage could be used as a functional food for the treatment and/or prevention of urolithiasis. However, further studies are required.

## References

[1] Alelign T Petros B. Kidney stone dise:ase: An update on current concepts. Adv Urol. 2018;2018:3068365. 10.1155/2018/3068365 29515627 PMC5817324

[2] Zampini A Nguyen AH Rose E Monga M Miller AW. Defining dysbiosis in patients with urolithiasis. Sci Rep. 2019;9(1):5425. 10.1038/s41598-019-41977-6 30932002 PMC6443657

[3] Stanford J Charlton K Stefoska-Needham A Ibrahim R Lambert K. The gut microbiota profile of adults with kidney disease and kidney stones: A systematic review of the literature. BMC Nephrol. 2020;21:215. 10.1186/s12882-020-01805-w 32503496 PMC7275316

[4] Vennila V Mariyal A. *In vitro* analysis of phytochemical and antiurolithiatic activity of various extracts of *Melia dubia* leaves. World J Pharm Pharm Sci. 2015;4(4):1277–89.

[5] Gnanandarajah JS Johnson T Kim H Abrahante J Lulich JP Murtaugh MP. Comparative faecal microbiota of dogs with and without calcium oxalate stones. J Appl Microbiol. 2012;113(4):745–56. 10.1111/j.1365-2672.2012.05390.x 22788835

[6] Gomathi S Sasikumar P Anbazhagan K Sasikumar S Kavitha M Selvi M Screening of indigenous oxalate degrading lactic acid bacteria from human faeces and South Indian fermented foods: Assessment of probiotic potential. ScientificWorldJournal. 2014;2014:648059. 10.1155/2014/648059 24723820 PMC3956639

[7] Hatch M. Gut microbiota and oxalate homeostasis. Ann Transl Med. 2017;5(2):36. 10.21037/atm.2016.12.70 28217701 PMC5300851

[8] Azcarate-Peril MA Bruno-Bárcena JM Hassan HM Klaenhammer TR. Transcriptional and functional analysis of oxalyl-coenzyme A (CoA) decarboxylase and formyl-CoA transferase genes from *Lactobacillus acidophilus.* Appl Environ Microbiol. 2006;72(3):1891–9. 10.1128/AEM.72.3.1891-1899.2006 16517636 PMC1393175

[9] Turroni S Vitali B Bendazzoli C Candela M Gotti R Federici F Oxalate consumption by lactobacilli: Evaluation of oxalyl‐CoA decarboxylase and formyl‐CoA transferase activity in *Lactobacillus acidophilus.* J Appl Microbiol. 2007;103(5):1600–9. 10.1111/j.1365-2672.2007.03388.x 17953571

[10] Okombo J Liebman M. Probiotic-induced reduction of gastrointestinal oxalate absorption in healthy subjects. Urol Res. 2010;38:169–78. 10.1007/s00240-010-0262-9 20224931

[11] Amini H Jahantigh M Galavi H Abdollahi A Pirouzi A Afkari R. Evaluation of oxalate-degrading activity and molecular Recognition of oxc, frc genes in lactic acid bacterium of inhabit in Human colon. Int J Pharm Technol. 2016;8(3):16055–66.

[12] Lieske JC. Probiotics for prevention of urinary stones. Ann Transl Med. 2017;5(2):29. 10.21037/atm.2016.11.86 28217694 PMC5300857

[13] Murru N Blaiotta G Peruzy MF Santonicola S Mercogliano R Aponte M. Screening of oxalate degrading lactic acid bacteria of food origin. Ital J Food Saf. 2017;6(2):6345. 10.4081/ijfs.2017.6345 28713789 PMC5505080

[14] Campieri C Campieri M Bertuzzi V Swennen E Matteuzzi D Stefoni S Reduction of oxaluria after an oral course of lactic acid bacteria at high concentration. Kidney Int. 2001;60(3):1097–105. 10.1046/j.1523-1755.2001.0600031097.x 11532105

[15] Chamberlain CA Hatch M Garrett TJ. Metabolomic profiling of oxalate-degrading probiotic *Lactobacillus acidophilus* and *Lactobacillus gasseri.* PLoS One. 2019;14(9):e0222393. 10.1371/journal.pone.0222393 31545840 PMC6756784

[16] Stepanova N Tolstanova G Akulenko I Savchenko O Lebid L Skovorodka M Kolesnyk M. Oxalate-degrading activity in fecal microbiota associated with blood lipid profile in dialysis patients. Nephrol Dial Transplant. 2020;35(Suppl 3):gfaa139.SO011. 10.1093/ndt/gfaa139.SO011

[17] Anbazhagan K Sasikumar P Gomathi S Priya H Selvam G. *In vitro* degradation of oxalate by recombinant *Lactobacillus plantarum* expressing heterologous oxalate decarboxylase. J Appl Microbiol. 2013;115(3):880–7. 10.1111/jam.12269 23734819

[18] Chakradhar K. A comparative clinical study on renal calculi – An ayurvedic perspective. J Med Dent Sci. 2012;2(5):21–32. 10.9790/0853-0252132

[19] Zeng Y Pu X Du J Yang X Li X Mandal M Molecular mechanism of functional ingredients in barley to combat human chronic diseases. Oxid Med Cell Longev. 2020;2020:3836172. 10.1155/2020/3836172 32318238 PMC7149453

[20] Shah JG Patel BG Patel SB Patel RK. Antiurolithiatic and antioxidant activity of *Hordeum vulgare* seeds on ethylene glycol-induced urolithiasis in rats. Indian J Pharmacol. 2012;44(6):672. 10.4103/0253-7613.103237 23248392 PMC3523490

[21] Sawicki CM McKay DL McKeown NM Dallal G Chen CYO Blumberg JB. Phytochemical pharmacokinetics and bioactivity of oat and barley flour: A randomized crossover trial. Nutrients. 2016;8(12):813. 10.3390/nu8120813 27983687 PMC5188468

[22] Sreeja V Patel K Prajapati JB. Oxalate degradation potential of lactic acid bacteria isolated from traditional fermented milk products, human vagina and human faecal matter. Int J Fermented Foods. 2018;7(2):125–9. 10.30954/2321-712X.12.2018.8

[23] IS 1479-1. Methods of test for dairy industry, Part 1: Rapid examination of milk. New Delhi, India: Bureau of Indian Standards; 1960. pp. 29-30. Available from: https://law.resource.org/pub/in/bis/S06/is.1479.1.1960.pdf.

[24] Chaudhary JK Mudgal S. Antidiabetic and hypolipidaemic action of finger millet (*Eleusine coracana*)-enriched probiotic fermented milk: An *in vivo* rat study. Food Technol Biotechnol. 2020;58(2):192–202. 10.17113/ftb.58.02.20.6308 32831571 PMC7416116

[25] Stone H, Bleibaum RN, Thomas HA, editors. Sensory evaluation practices - Food science and technology. Amsterdam, The Netherlands: Elsevier; 2004.

[26] Manual of methods of analysis of foods. New Delhi, India: Food Safety and Standards Authority of India (FSSAI); 2015. pp. 34-40, 49-50. Available from: https://www.fssai.gov.in/upload/uploadfiles/files/milk_and_milk_products.pdf.

[27] IS 1479-2. Method of test for dairy industry, Part 2: Chemical analysis of milk. New Delhi, India: Bureau of Indian Standards; 1961. pp. 28-29. Available from: https://law.resource.org/pub/in/bis/S06/is.1479.2.1961.pdf.

[28] Hodgkinson A. A combined qualitative and quantitative procedure for the chemical analysis of urinary calculi. J Clin Pathol. 1971;24(2):147–51. 10.1136/jcp.24.2.147 5551382 PMC476935

[29] Asano I Ikeda Y Fujii S Lino H. Effects of mannooligosaccharides from coffee on microbiota and short chain fatty acids in rat cecum. Food Sci Technol Res. 2007;10(3):273–7. 10.3136/fstr.10.273

[30] GraphPad Prism, v. 8.00, GraphPad Software Inc., San Diego, CA, USA; 2018. Available from:https://www.freesoftwarefiles.com/education/graphpad-prism-8-0-free-download/

[31] Murphy C Murphy S O’Brien F O’Donoghue M Boileau T Sunvold G Metabolic activity of probiotics – Oxalate degradation. Vet Microbiol. 2009;136(1-2):100–7. 10.1016/j.vetmic.2008.10.005 19028028

[32] Karadi RV Gadge NB Alagawadi KR Savadi RV. Effect of *Moringa oleifera Lam*. Root-wood on ethylene glycol induced urolithiasis in rats. J Ethnopharmacol. 2006;105(1-2):306–11. 10.1016/j.jep.2005.11.004 16386862

[33] Makasana A Ranpariya V Desai D Mendpara J Parekh V. Evaluation for the anti-urolithiatic activity of *Launaea procumbens* against ethylene glycol-induced renal calculi in rats. Toxicol Rep. 2014;1:46–52. 10.1016/j.toxrep.2014.03.006 28962225 PMC5598485

[34] Khan SR Johnson JM Peck AB Cornelius JG Glenton PA. Expression of osteopontin in rat kidneys: Induction during ethylene glycol induced calcium oxalate nephrolithiasis. J Urol. 2002;168(3):1173–81. 10.1016/S0022-5347(05)64621-6 12187263

[35] Khan SR Shevock PN Hackett RL. Acute hyperoxaluria, renal injury and calcium oxalate urolithiasis. J Urol. 1992;147(1):226–30. 10.1016/S0022-5347(17)37202-6 1729537

[36] Cunningham P Noble H Al-Modhefer AK Walsh I. Kidney stones: Pathophysiology, diagnosis and management. Br J Nurs. 2016;25(20):1112–6. 10.12968/bjon.2016.25.20.1112 27834524

[37] Fan J Glass MA Chandhoke PS. Impact of ammonium chloride administration on a rat ethylene glycol urolithiasis model. Scanning Microsc. 1999;13(2-3):299–306.

[38] Barnela SR Soni SS Saboo SS Bhansali AS. Medical management of renal stone. Indian J Endocrinol Metab. 2012;16(2):236. 10.4103/2230-8210.93741 22470860 PMC3313741

[39] Grover PK Resnick MI. Evidence for the presence of abnormal proteins in the urine of recurrent stone formers. J Urol. 1995;153(5):1716–21. 10.1016/S0022-5347(01)67511-6 7715017

[40] Ahmed S Hasan MM Mahmood ZA. *In vitro* urolithiasis models: An evaluation of prophylactic management against kidney stones. J Pharmacogn Phytochem. 2016;5(3):28–35.

[41] Wesson JA Johnson RJ Mazzali M Beshensky AM Stietz S Giachelli C Osteopontin is a critical inhibitor of calcium oxalate crystal formation and retention in renal tubules. J Am Soc Nephrol. 2003;14(1):139–47. 10.1097/01.ASN.0000040593.93815.9D 12506146

[42] Fernandez M, Picard-Deland É, Le Barz M, Daniel N, Marette A. Yogurt and health. In: Frias J, Martinez-Villaluenga C, Penas E, editors. Fermented foods in health and disease prevention. Amsterdam, The Netherlands: Elsevier; 2017. pp. 305-38. 10.1016/B978-0-12-802309-9.00013-3 10.1016/B978-0-12-802309-9.00013-3

[43] Taheri H Miri A Bokaeian M Cambyz I Afkari R. The evaluation of lithiasis/antilithiatic activity of oxalate-degrading bacteria and routine antibiotics. Ann Rom Soc Cell Biol. 2021;25(4):16514–22.

[44] Hirose M Yasui T Okada A Hamamoto S Shimizu H Itoh Y Renal tubular epithelial cell injury and oxidative stress induce calcium oxalate crystal formation in mouse kidney. Int J Urol. 2010;17(1):83–92. 10.1111/j.1442-2042.2009.02410.x 19919640

[45] Riane K Sifour M Ouled-Haddar H Espinosa C Esteban MA Lahouel M. Effect of probiotic supplementation on oxidative stress markers in rats with diclofenac-induced hepatotoxicity. Braz J Microbiol. 2020;51:1615–22. 10.1007/s42770-020-00302-4 32458261 PMC7688739

[46] Liu Y Jin X Hong HG Xiang L Jiang Q Ma Y The relationship between gut microbiota and short chain fatty acids in the renal calcium oxalate stones disease. FASEB J. 2020;34(8):11200–14. 10.1096/fj.202000786R 32645241

[47] Jin X Jian Z Chen X Ma Y Ma H Liu Y Short chain fatty acids prevent glyoxylate-induced calcium oxalate stones by GPR43-dependent immunomodulatory mechanism. Front Immunol. 2021;12:729382. 10.3389/fimmu.2021.729382 34675921 PMC8523925

[48] Petrova P Petrov K. Lactic acid fermentation of cereals and pseudocereals: Ancient nutritional biotechnologies with modern applications. Nutrients. 2020;12(4):1118. 10.3390/nu12041118 32316499 PMC7230154

[49] Lambo AM Öste R Nyman MEL. Dietary fibre in fermented oat and barley β-glucan rich concentrates. Food Chem. 2005;89(2):283–93. 10.1016/j.foodchem.2004.02.035

[50] Arena MP Caggianiello G Fiocco D Russo P Torelli M Spano G Barley β-glucans-containing food enhances probiotic performances of beneficial bacteria. Int J Mol Sci. 2014;15(2):3025–39. 10.3390/ijms15023025 24562330 PMC3958897

[51] García-Burgos M Moreno-Fernández J Alférez MJ Díaz-Castro J López-Aliaga I. New perspectives in fermented dairy products and their health relevance. J Funct Foods. 2020;72:104059. 10.1016/j.jff.2020.104059

